# Relation between Inflammation, Oxidative Stress, and Macronutrient Intakes in Normal and Excessive Body Weight Adolescent Girls with Clinical Features of Polycystic Ovary Syndrome

**DOI:** 10.3390/nu13030896

**Published:** 2021-03-10

**Authors:** Małgorzata Mizgier, Grażyna Jarząbek-Bielecka, Natalia Wendland, Elżbieta Jodłowska-Siewert, Marcin Nowicki, Alicja Brożek, Witold Kędzia, Dorota Formanowicz, Justyna Opydo-Szymaczek

**Affiliations:** 1 Department of Dietetics, Faculty of Physical Culture in Gorzów Wlkp., Poznan University of Physical Education, Estkowskiego 13, 66-400 Gorzów Wielkopolski, Poland; 2 Department of Perinatology and Gynecology, Division of Developmental Gynecology and Sexology, Poznan University of Medical Sciences, 60-535 Poznan, Poland; grajarz@tlen.pl (G.J.-B.); witold.kedzia@poczta.fm (W.K.); 3 Department of Pediatric Dentistry, Chair of Pediatric Dentistry, Poznan University of Medical Sciences, 60-812 Poznan, Poland; nataliakryszan@interia.pl (N.W.); jopydo@ump.edu.pl (J.O.-S.); 4 Department of Computer Science and Statistics, Poznan University of Medical Sciences, 60-806 Poznan, Poland; youleadmeastray@gmail.com; 5 Chair and Department of Medical Chemistry and Laboratory Medicine, Poznan University of Medical Sciences, 60-806 Poznan, Poland; nowickim@ump.edu.pl (M.N.); abrozek@ump.edu.pl (A.B.); doforman@ump.edu.pl (D.F.)

**Keywords:** polycystic ovary syndrome, adolescent girls, obesity, diet, food environments, hyperandrogenism

## Abstract

The impact of diet on inflammation and oxidative stress (OS) in girls with polycystic ovary syndrome (PCOS) is unknown. Therefore, our study aimed to investigate, in PCOS girls, whether certain macronutrient intakes can be associated with these disturbances. For this purpose, 59 PCOS participants (aged 14–18 years) were recruited to this study and divided into two subgroups: overweight/obese—Ov/Ob group (*n* = 22) and normal weight—N group (*n* = 37). Nutrition was assessed using a 3-day food record. The studied markers were total antioxidant capacity (TAC), malondialdehyde (MDA), C-reactive protein (CRP), tumor necrosis factor α (TNF-α), and interleukins 1 and 6 (IL-1 and IL-6). We found plant protein intake inversely correlated with IL-6 (*p* = 0.007; *r* = −0.557), TNF-α (*p* = 0.006; *r* = −0.564), MDA (*p* = 0.01; *r* = −0.539) in the Ov/Ob group and with TAC (*p* = 0.021; *r* = −0.38) in the N group. Inverse correlations in the Ov/Ob group were observed between protein intake and IL-6 (*p* = 0.031; *r* = −0.461), TNF- α (*p* = 0.043; *r* = −0.435); carbohydrates and IL-6 (*p* = 0.037; *r* = −0.448), MDA (*p* = 0.045; *r* = −0.431); fiber and IL-6 (*p* = 0.025; *r* = −0.475). A positive relationship between cholesterol intake and CRP concentration (*p* = 0.038; *r* = 0.342) was also found in the N group. These findings revealed that inflammation and OS are increased in Ov/Ob girls with decreased plant protein intake and low carbohydrates in the diet. Moreover, inflammation may be increased by cholesterol intake in slim PCOS girls. On the other hand, decreased intake of fiber and total protein intake increased inflammation. ClinicalTrials.gov Identifier: NCT04738409.

## 1. Introduction

Many research studies indicate that metabolic changes in the human body are associated with a low-degree chronic inflammatory process [1,2] coexisting with oxidative stress (OS) [3,4]. Both disorders underlie many chronic conditions, including those related to diet [2,5,6,7]. It seems that such a disease is polycystic ovary syndrome (PCOS) [8,9,10,11,12]. Hyperandrogenism, the hallmark of PCOS, is clinically manifested by acne, masculinization, and higher incidence of metabolic disorders such as insulin resistance (IR); dyslipidemia, mainly visceral obesity; arterial hypertension; and type 2 diabetes mellitus [13,14]. The metabolic and hormonal profile is more severe in PCOS girls with excess body weight and overweight than normal-weighted. Obesity is observed in 40–70% of young women with PCOS [6,7,15,16,17,18,19,20,21,22]. The prevalence of PCOS in adolescent girls based on a meta-analysis covering 149,477 patients from different countries (India, Iran, Thailand, Australia, and USA) is 8.67%. Depending on the diagnostic criteria, the frequency is 11.04% (based on the Rotterdam criteria), 3.39% (based on the National Institutes of Health criteria), and 8.03% (based on Androgen Excess and Polycystic Ovary Syndrome Society criteria) [17].

Women with PCOS of childbearing age show elevated levels of inflammatory markers in the serum, including C-reactive protein (CRP); interleukins (IL)—IL-1, IL-6, and IL-18; and tumor necrosis factor α (TNF-α) compared with healthy women [23,24]. The CRP increase can stimulate the liver and monocytes to increase the proinflammatory factors’ secretion, ultimately causing an increased insulin resistance from insulin-sensitive tissue [2,25,26,27,28]. Ren, Z. et al. showed that elevated CRP is associated with increased waist circumference (WC), systolic and/or diastolic blood pressure (SBP and/or DBP, respectively), fasting glucose and triglycerides (TG), and decreased high-density lipoprotein cholesterol (HDL-C) [2]. Patients with metabolic disorders may also have significantly higher IL-6 and TNF-α levels than healthy controls [29]. As previously mentioned, OS may also contribute to the presence of low-grade systemic chronic inflammation [3,30]. OS, as a disruption of homeostasis between prooxidants and antioxidants activity, results in damage to biologically important molecules. Inflammatory processes induce oxidative stress, while oxidative stress, causing injury to healthy cells, triggers inflammation [31,32]. Several exogenous dietary factors may act as prooxidants by increasing reactive oxygen species (ROS). For example, it may increase OS by catalyzing highly reactive hydroxyl radicals via the Haber–Weiss reaction [33]. Exposure to oxidants can lead to inflammation and subsequent endothelial dysfunction, with eventual cardiovascular disease ensuing [21,34,35,36].

Increased OS and inflammation are both strongly associated with obesity in adults [8,37]. The relationship between childhood obesity and inflammation is also confirmed, but data on childhood obesity and OS are emerging [1,38]. Obesity induces chronic low-grade inflammation [11,12,38,39]. These processes are most likely related to the adipocyte’s abnormal function and the dysregulation of adipocytokines secreted by adipose tissue [12,39,40].

The increased load of ROS typical for OS induces the activation of transcription factors, for example, NF-κB, which is involved in the formation of inflammation in cells (interacts with the production of proinflammatory cytokines such as TNF-α and IL-6) [41]. The initial activation of the systemic inflammatory response in plasma observed in PCOS is associated with increased ROS production. It is due to mitochondrial damage and dysfunction. It should be noticed that mitochondria play a vital role in developing OS and further activating the inflammatory response. Excessive production of ROS leads to a compensatory increase in the activity of non-enzymatic and enzymatic antioxidants [14,42,43].

As we already know, diet is a common mediator between inflammation and the development of chronic diseases [44,45,46]. To develop effective prophylactic and therapeutic strategies for patients in the early stages of PCOS, it is vital to understand whether and how individual dietary components affect the immune system functioning, the inflammation and OS associated with PCOS. The studies conducted so far indicate that higher values of the Dietary Inflammatory Index (DII), which is also observed in people with metabolic and inflammatory disorders [47,48,49,50,51]. The DII was developed to assess the overall inflammatory potential of a diet. In a study conducted by Shivappa et al., the following macronutrients: carbohydrates, fiber, protein, total fat, saturated fat, monounsaturated fat, polyunsaturated fat, and cholesterol were used to calculate the DII [51]. Shivappa et al. demonstrated that a proinflammatory diet with a higher DII is significantly associated with an increased CRP [41,51]. A proinflammatory diet, along with a sedentary lifestyle, contributes to a chronic low-grade inflammatory process, orchestrated by metabolic cells in response to excess energy and nutrients, known as metaflammation. It is a growing public health problem with global epidemic dimensions [2,4,52]. It has been proven that the impact of ROS- and OS-induced inflammation can be reversed by certain antioxidant nutrients [3,53,54,55,56,57,58,59].

Our study’s objective was to investigate whether normal and overweight/obese girls with PCOS clinical features differ in their inflammatory or OS status based on selected biomarkers’ assessment. Moreover, we would like to check if there is a relationship between OS, inflammatory biomarkers, and macronutrients intake in PCOS girls.

## 2. Materials and Methods

### 2.1. Participants and Ethical Aspects

Fifty-nine Caucasian adolescents aged 14–18 years participated in the study. The girls were recruited using convenience sampling methods among the Medical Clinic of Gynecology and Perinatology patients at the Gynecology and Obstetrics Hospital of Poznan University of Medical Sciences. Within the study period (from 2018 to 2020), all patients who met the eligibility criteria were included. The participants were enrolled in the study at the time of diagnosis. The girls were divided into two groups: the N group consisting of 37 normal-weight patients (mean age ± standard deviation (SD) = 16.35 ± 1.14) and the Ov/Ob group of 22 young females who were overweight or obese (mean age ± SD = 15.77 ± 1.6).

The study’s inclusion was based on the 2003 Rotterdam PCOS diagnostic criteria (clinical and/or biochemical hyperandrogenism; oligo-/amenorrhea; polycystic ovary image in an ultrasound examination).

Exclusion criteria included any systemic diseases, thyroid dysfunction, diabetes mellitus, congenital adrenal hyperplasia, Cushing’s syndrome, hyperprolactinemia suggestive of pituitary adenoma and androgen-secreting tumors or drugs in continuous use, use of hormone therapy or antibiotics in the last three months, intake of vitamins or supplements, drinking alcohol, and smoking. The eligibility criteria of the research have previously been described in detail [22,60,61].

The Bioethics Committee at the Poznan University of Medical Sciences approved this cross-sectional study (resolution no. 553/18). All young females and their parents gave their informed consent for inclusion before participating in the study.

This study was registered retrospectively at ClinicalTrials.gov (accessed on 4 February 2021) (ClinicalTrials.gov Identifier: NCT04738409) as registration was not required when study enrolment started. The authors confirm that all ongoing and related trials associated with this study are registered.

### 2.2. Medical Evaluation

During a two-day hospital stay, the patients underwent a gynecological consultation in the outpatient department. The gynecologist (G.J.B.) detected the presence of any symptoms of hormonal disturbances based on the patient’s history (menstrual cycle regularity and age of menarche), the physical examination of the skin (for signs of virilization, like hirsutism, and acne), blood tests, and a transabdominal ultrasound examination [62].

Anthropometric and body composition measurements, including height, body mass, and waist circumference (WC), were conducted after a 12-h fast.

Overweight and obesity were diagnosed and classified based on body mass index (BMI). For children aged 5–19 years, according to the definition of the World Health Organization (WHO), overweight and obesity correspond to a BMI-for-age higher than 1 and 2 SD above the growth reference median, respectively [63]. The tables with BMI-for-age z-score values from −3 to +3 SD displayed by age (years and months) are available on the WHO website (http://www.who.int/tools/growth-reference-data-for-5to19-years/indicators/bmi-for-age (accessed on 21 December 2020)).

Body composition was evaluated with Bioelectrical Impedance Analyzer (BIA; Tanita MC780). Fat mass (FM) measurements were expressed as percentages (%) and kilograms (kg). Detailed information has been provided elsewhere [64,65].

The procedure of medical, anthropometric, and Body Composition Assessment has previously been described in detail in [22].

### 2.3. Nutritional Evaluation

To assess eating habits, a registered dietitian (M.M.) used the method named the “three-day food record” [66]. In this method, the patient records the amount of all meals consumed and food products needed to prepare the meals. The participants filled in the record after each meal during two business days and one holiday day (three consecutive days). To assess the size of the portion consumed, girls used an album of photos of the products and dishes [67]. Daily food rations’ energy and nutritional value were calculated using the Cambridge Diagnostics computer program “Aliant”.

The analyzed daily food intake of all patients (taking into account standard losses because of technical and culinary treatment) was compared with the current, individual daily nutritional and energy requirement. Each patient’s energy and the nutritional requirement was determined at the recommended level based on the Human Nutrition Standards of the National Food and Nutrition Institute [68].

The procedure for nutritional evaluation has previously been described in detail [22].

### 2.4. Biochemical Parameters

Blood tests and a gynecological examination with ultrasound studies were carried out in the early follicular phase on the 3rd to 5th day (apart from patients reporting secondary amenorrhea).

Biochemical and hormonal parameters, including luteinizing hormone (LH), follicle-stimulating hormone (FSH), sex hormone-binding globulin (SHBG), 17-β-estradiol, total testosterone, DHEA-S, insulin, glucose, triglycerides (TG), total cholesterol (TC), and (HDL-C), were measured in the morning, after overnight fasting. The analyses were performed in the central laboratory of the Gynecology and Obstetrics Hospital of Poznan University of Medical Sciences. Insulin, LH, FSH, SHGB, total testosterone, 17-β-estradiol, and DHEA-S were analyzed by the electrochemiluminescence (ECLIA) immunoassay method (Elecsys) (Roche Diagnostics GmbH, Mannheim, Germany). Plasma TC, HDL-C, and TG levels were determined using the enzymatic colorimetric method (Roche Diagnostics GmbH, Mannheim, Germany). Low-density lipoprotein cholesterol (LDL-C) concentration was calculated using Friedewald’s formula: LDL-C (mg/dL) = TC − HDL-C − (TG/5). Plasma glucose was assessed by the enzymatic method with hexokinase.

Homeostasis model assessment-IR (HOMA-IR) was applied using the following formula: HOMA-IR = fasting insulin (μU/mL) × fasting glucose (mmol/L)/22.5 [69,70].

Biochemical analyses of oxidative and inflammatory markers were performed by medical lab analysts (A.B, M.N.) in the Chair and Department of Medical Chemistry and Laboratory Medicine, Poznan University of Medical Sciences. The samples tested were marked in doublet.

Serum concentrations of the selected inflammatory markers (IL-6, IL-1, TNF-α, and CRP), and malondialdehyde (MDA), an oxidative stress marker, were measured with a commercial assay kit. The serum markers concentration was determined by means of SunRed tests with the Enzyme-Linked Immunosorbent Assay (ELISA) method using the TECAN-SUNRISE reader with the Magellan software. The kit uses monoclonal antibodies directed against the antigenic determinants of the analyte being analyzed. Total antioxidant capacity (TAC) was measured with a commercial assay kit (Omnignostica Forschungs GmbH, St. Andrä-Wördern, Austria). Both small molecule and protein antioxidants were measured, and the results were presented as a mmol/L Trolox equivalent.

### 2.5. Statistical Analyses

Statistical analysis was conducted using PQStat v1.8.0 software. The Shapiro–Wilk test was used to verify whether quantitative data were distributed normally. The Fisher–Snedecor test was used to check if the variances of the groups being compared were significantly different. When comparing two groups, the unpaired *t*-test was used for normally distributed quantitative data (with Cochran–Cox correction if the groups’ variances in question were significantly different), and the Mann–Whitney test for not normally distributed or ordinal data. When assessing correlations between data, the Pearson correlation coefficient was calculated for normally distributed variables. Spearman’s rank correlation coefficient was used for ordinal data and when the assumption of normality was not met. Descriptive statistics of groups were presented as mean and standard deviation, median and quartiles, as well as numbers and percentages, depending on the data type.

A *p*-value of 0.05 was the level of statistical significance in all analyses.

## 3. Results

### 3.1. Anthropometric, Clinical, and Metabolic Parameters

Overweight and obese girls (Ob/Ov group) had significantly higher fat mass expressed as a percentage (FM%) (*p* < 0.000001) and kilograms (FMkg) (*p* = 0.000001) as compared to their slim counterparts. In the Ob/Ov group, a significantly higher SBP (*p* = 0.00001) and DBP (*p* = 0.004), WC (*p* < 0.000001), and other metabolic parameters such as TG (*p* < 0.047) and fasting insulin (*p* = 0.0015) concentration, were seen. The HDL-C concentration was significantly lower (*p* = 0.00009). HOMA-IR was significantly higher (*p* = 0003), and the Quicki index was significantly lower (*p* = 0.0007) in the Ov/Ob group (Table 1).

### 3.2. Markers of Inflammation and Oxidation

The mean and median concentrations of the studied OS and inflammatory markers seem to be higher, and only TAC appears to be lower in the Ov/Ob group than in the N group; however, these differences are not statistically significant (Table 2).

We also observed that in the Ov/Ob group, plant protein intake was inversely correlated with oxidative markers (MDA: *r* = −0.54, *p* = 0.01) and with markers of inflammation (IL-6: *r* = −0.56, *p* = 0.007; TNF-α: *r* = −0.56, *p* = 0.006) (Table 3 and Table 4). Plant protein intake was also inversely correlated with TAC, but only in the N group (*r* = −0.38, *p* = 0.02) (Table 4 and Table 5).

No relationship was found between animal protein intake and the analyzed markers, but there was a significant negative relationship between total protein intake and IL-6 (*r* = −0.46, *p* = 0.03) and TNF-α (*r* = −0.43, *p* = 0.04) in the Ov/Ob group (Table 3 and Table 4).

Total carbohydrate intake was inversely correlated with IL-6 (*r* = −0.45, *p* = 0.04), MDA (*r* = −0.43, *p* = 0.05) and fiber intake with IL-6 (*r* = −0.48, *p* = 0.04) in the Ov/Ob group (Table 3 and Table 4).

No significant relationship was found between the inflammation and OS markers tested and intake of total fat, saturated fat (SFA), polyunsaturated fat (PUFA), and monosaturated fat (MUFA). However, a significantly positive relationship between cholesterol intake and CRP (*r* = 0.34, *p* = 0.04) in the N group was revealed (Table 4 and Table 5).

## 4. Discussion

PCOS is a heterogeneous disease associated with metabolic disturbances, OS, and chronic systemic low-grade inflammation [3,10,71,72,73,74,75,76,77,78,79,80]. It is still unclear to what extent oxidative and inflammatory changes in patients with PCOS result from the disease itself or the sequelae of metabolic syndrome.

As previously proven, obesity increases the low-grade chronic inflammation associated with OS in the body [81,82]. It is related to abnormalities in adipocytes and dysregulation of adipocytokines secreted by adipose tissue [83]. In the study by Block G. et al., increased body weight in adults was associated with an increase in OS [84]. Moreover, a survey conducted by Montero D. et al. also confirmed in children the existence of the relationship between obesity and OS [81].

In adult patients with PCOS, systemic inflammatory activation and mitochondrial dysfunction may occur regardless of high BMI and the coexistence of metabolic diseases [9,10,85,86,87,88]. However, opposite results were reported by Mohlig et al., who revealed that neither CRP nor IL-6 was significantly elevated in slim or obese PCOS women compared with age-matched slim or obese controls. In contrast, insulin resistance, BMI, and waist to hip ratio correlated with inflammation markers [89].

Similarly, a recent study by Khashchenko et al. involving 95 girls with PCOS and 30 healthy, lean age-mates revealed that PCOS was not an independent factor of increased levels of CRP, while high BMI showed a significant impact on CRP level. This study did not confirm any activation of systemic inflammation and OS in individuals with PCOS, normative BMI and impaired glucose tolerance [88]. However, these researchers disclosed a significant increase in the anti-inflammatory cytokine—interleukin 10 (IL-10) and decreased lipid peroxidation (LPO) marker in the study group, probably due to the activation of the homeostasis control system and adaptive compensation of the antioxidant defense mechanisms [88]. The authors assert that overweight and obesity in girls with PCOS may reduce the efficiency of antioxidant systems. Besides, excess weight, reduction of glucose utilization, and dyslipidemia with fatty acids accumulation in tissues can activate OS and inflammatory response [88]. The results emphasize the need for early intervention in the group with overweight aimed at the BMI reduction and improvement of carbohydrate metabolism, which may impede the progression of low-grade chronic systemic inflammation. The proposed preventive strategy includes a restrictive diet, physical exercises, and insulin sensitizers combined with supplements that improve mitochondrial functioning [88].

In our study in girls with PCOS, the MDA concentration and the inflammatory markers studied, like CRP, TNF-α, IL-1, and IL-6, were higher in the Ob/Ov group, but the differences were non-statistically significant. The total value of TAC appeared to be slightly higher in the N group, but we did not note any significant differences between both groups in this respect. However, our Ob/Ov group comprised both girls with IR and girls with normal carbohydrate metabolism, so contrary to Khashchenko et al. [88], we could not observe independent effects of overweight and metabolic disturbances on the OS and inflammatory status.

It is known that a well-balanced diet might have a positive impact on the metabolic profile of females affected by PCOS. Toscani M.K. et al. indicated in their intervention study that a more significant reduction of body weight and fat tissue was achieved in adult patients with PCOS following a diet high in whey protein [90]. Our previous study, involving the same group of individuals with PCOS, showed that the likelihood of being overweight or obese was halved by an increase in plant protein intake of 10 g/day [22]. However, it has not been established whether the development of inflammation and OS in girls with PCOS might be influenced by the ratio and amount of macronutrients intake. Therefore, our study aimed to check whether the levels of OS and inflammation markers in adolescent PCOS patients correlate with the consumption of proteins, carbohydrates and fiber, fats, and cholesterol. We investigated the above correlations separately in patients with normal weight and excess body weight, as the metabolic profile differs in overweight and obese PCOS patients compared to slim ones [6,7,15,16,17,18,19,20,21,22].

Likely, OS and inflammation in girls with PCOS are additionally promoted by diet. Poor nutrition can trigger OS and inflammation in the body [2,3,4,5,51,91] and affect levels of inflammatory markers such as CRP [41,51]. It has been shown that proinflammatory dietary components can induce a chronic metabolic inflammation state [2,4,52]. However, the effects of ROS- and OS-induced inflammation can be reversed by certain antioxidant nutrients [3,53,54,55,56,57,58,59].

In the Ob/Ov group, we observed a significant inverse correlation between total protein consumption, plant protein, and the concentration of proinflammatory cytokines: IL-6, TNF-α. Moreover, we found the inverse relationship between plant protein consumption and MDA (lipid peroxidation marker). The findings from other studies also confirm that protein intake is inversely related to inflammation and OS [92,93,94,95,96].

Our current research results indicate that higher carbohydrate consumption in overweight and obese girls was associated with a lower concentration of IL-6 and MDA. In turn, lower fiber consumption was associated with a higher IL-6 concentration.

Khayyatzadeh et al. also showed an inverse correlation between the consumption of carbohydrates and dietary fiber and the occurrence of inflammation, expressed in the concentration of the hs-CRP marker in the serum [97]. Lower fiber content is characteristic for products with a high glycemic index (GI), while products with low GI usually have higher dietary fiber content [98]. A systematic review of interventional and observational studies by Buyken A.E. et al. showed that lower concentrations of hsCRP or IL-6 are associated with a higher intake of fiber and carbohydrates with a lower GI and glycemic load (GL) [99]. In a dietary intervention carried out among adult women with PCOS, Szczuko M. et al. showed that the consumption of carbohydrates with low GI/GL, high fiber content is associated with a reduction in inflammation in the body [100]. Low-fiber carbohydrate intake with a high GI causes acute postprandial hyperglycemia, which increases inflammation and OS. Barrea et al. among women with PCOS, showed that high carbohydrate consumption and low-grade inflammation coexisting with IR and hyperandrogenism contributing to the pathogenesis of PCOS. In regulating the activity of ovarian and hepatic enzymes involved in androgen production and inducing low-grade inflammation associated with IR, dyslipidemia, and cardiometabolic diseases, insulin’s role is crucial in PCOS [101].

Note that most correlations between diet and markers of inflammation and OS in studied patients were disclosed in PCOS girls with excess body weight. On the other hand, in girls with normal body weight, we only observed that the increase in cholesterol consumption was related to CRP concentration.

A significant positive relationship between dietary cholesterol and hsCRP concentration was found by Khayyatzadeh et al. [97]. The other studies discovered that eggs’ cholesterol content possesses proinflammatory properties by inducing cytotoxicity and stimulating lipid rafts’ formation in leukocytes’ plasma membranes, resulting in increased sensitivity to proinflammatory signaling [102,103].

The relationship between dietary cholesterol intake and CRP and other proinflammatory cytokines was not confirmed in our study in the group of girls with excess body weight. In the Ob/Ov group, we also did not find the inverse relationship between plant protein consumption and TAC concentration, which was revealed in the normal weight PCOS girls.

Barbosa et al. found that in healthy and predominantly eutrophic adults, daily macronutrient intake, including protein intake, is inversely correlated with plasma TAC. They hypothesize that with decreased accessibility of oxidizable substrates, the recruitment of enzymatic defense systems is diminished, consequently preserving high plasma TAC [104]. It remains a matter of debate whether this would explain the negative correlation between plant protein intake and TAC observed in our N group individuals. Another possible reason for this inverse relationship is that the TAC was modulated in our slim patients by other environmental factors (so-called disruptors), which reduced antioxidant systems’ efficiency, such as passive smoking or stress. None of the girls surveyed smoked tobacco, but unfortunately we did not collect passive smoking and stress exposure data. It must be underlined that diminished TAC in patients with PCOS is disadvantageous, leading to a reduction in antioxidant defense [105].

As was already mentioned, we found most correlations between dietary macronutrients (protein, carbohydrate, and fiber intake) and markers of inflammation and OS in girls with excess body weight. It emphasizes the role of a well-balanced diet in the prevention of OS and inflammation in this group.

### Strengths and Weaknesses

The study has some limitations that should be taken into account when interpreting the obtained results. The PCOS diagnosis was based on the Rotterdam criteria [60,61], while some authors propose stricter criteria for young girls as the symptoms of puberty can mimic those typical of endocrinopathy [106]. We did not collect data on some potential environmental factor implications that may modulate inflammation and OS in young women [71]. These factors include passive exposure to tobacco smoke and stress. Besides, our computer program for calculating the nutritional value of daily food rations did not include anti- and prooxidative ingredients, such as flavones or trans-fatty acids.

However, it must be noted that, despite its cross-sectional nature and relatively small sample, it is the first study that confirms a relationship between the intake of dietary macronutrients, inflammation, and OS in young females with PCOS.

## 5. Conclusions

Our study results show a relationship between eating habits and the occurrence of inflammation and OS in adolescent girls with PCOS clinical features, especially those who are overweight and obese.

Our findings showed that total and/or plant protein, carbohydrates, and fiber intake were inversely associated with the inflammation and/or OS markers. We did not show a significant relationship between fat intake and the markers measured, but cholesterol intake adversely correlated with hsCRP concentration in slim girls with PCOS.

Further studies are required to explain the relationship between diet and hormonal abnormalities, inflammation, and OS.

## Figures and Tables

**Table 1:** Comparison of the baseline parameters including age, anthropometric, and metabolic parameter characteristics between groups.

Variables	N Group *n* = 37	Ov/Ob. Group *n* = 22	*p*-Value
Age (years)			0.211 *
Mean (SD)	16.35 (1.14)	15.77 (1.6)	
Median(25–75%)	16 (16−17)	16 (14.25−17)	
Body height (m)			0.604
Mean(SD)	1.66 (0.07)	1.67 (0.05)	
Median(25–75%)	1.65 (1.61−1.71)	1.67 (1.64−1.69)	
Body weight (kg)			**<0.000001**
Mean(SD)	55.61 (7.13)	83.66 (11.76)	
Median (25–75%)	54.7 (50.9−62.1)	83.05 (74.95−92.18)	
Fat mass (%)			**<0.000001**
Mean(SD)	20.41 (6.72)	34.18 (6.89)	
Median (25–75%)	19.2 (16.6–26.1)	33.5 (29.58−39.53)	
Fat mass (kg)			0.000001 *
Mean(SD)	8.41 (5.09)	20.64 (9.99)	
Median (25–75%)	7.4 (4.8−10.2)	17.85 (12.9−25.85)	
SBP (mmHg)			**0.00001**
Mean(SD)	105.22 (7.87)	117.05 (10.82)	
Median (25–75%)	106 (99−110)	115.5 (111.25−123.75)	
DBP (mmHg)			**0.004**
Mean(SD)	68.59 (8.36)	75.5 (9)	
Median(25–75%)	68 (62−76)	77 (68.75−81)	
Waist Circumference (WC) (cm)			**<0.000001**
Mean(SD)	71.41 (6.32)	92.09 (9.47)	
Median(25–75%)	70 (67−76)	91 (86.25−96.5)	
TC (mg/dL)			0.520 *
Mean(SD)	156.38 (30.6)	157.77 (22.64)	
Median(25–75%)	157.7 (135.2−169.6)	162.35 (137.05−176)	
LDL-C (mg/dL)			0.108 *
Mean(SD)	79.78 (27.35)	86.36 (19.39)	
Median(25–75%)	79.2 (57.9−93.6)	86 (72.1−101.38)	
HDL-C (mg/dL)			**0.00009**
Mean(SD)	58.88 (10.72)	49.44 (6.42)	
Median (25–75%)	57.7 (52.3−67.4)	48.3 (44.78−53.38)	
TG (mg/dL)			0.047 *
Mean(SD)	87.71 (34.4)	109.9 (41.69)	
Median(25–75%)	83.7 (62.2−109.6)	100.55 (74.55–148.13)	
Fasting glucose (mg/dL)			0.138
Mean(SD)	87.59 (5.89)	90.3 (7.65)	
Median(25–75%)	87.8 (84.5−91.1)	89.6 (85.8−96.9)	
Fasting insulin (mU/mL)			0.0015 *
Mean(SD)	13.12 (6.95)	19.76 (8.75)	
Median (25–75%)	11.54 (8.73−14.96)	18.63 (14.88−26.85)	
HOMA-IR			0.0003 *
Mean (SD)	2.82 (1.72)	4.72 (2.36)	
Median(25–75%)	2.5 (1.79−3.28)	4.3 (3.26−6.37)	
Quicki			0.0007 *
Mean(SD)	0.33 (0.03)	0.31 (0.03)	
Median(25–75%)	0.33 (0.32−0.35)	0.31 (0.29−0.32)	

SBP—systolic blood pressure [mmHg]; DBP—diastolic blood pressure [mmHg]; TC—total cholesterol, LDL-C—low-density lipoprotein cholesterol; HDL-C—high-density lipoprotein cholesterol; TG—triglycerides, HOMA-IR—Homeostatic Model Assessment of Insulin Resistance. The analysis was conducted using an unpaired *t*-test (not marked) or Mann–Whitney test (*p*-value marked with an asterisk). All the *p*-values marked in bold were statistically significant.

**Table 2:** Comparison of the inflammatory and antioxidant markers between groups.

Variables	N Group *n* = 37	Ov/Ob. Group *n* = 22	*p*-Value
IL-1 (pg/mL)			0.216 *
Mean(SD)	27.71 (14.58)	44.03 (57.76)	
Median(25–75%)	24.7 (17.34–31.04)	26.27 (21.51–34.14)	
IL-6 (ng/L)			0.297 *
Mean(SD)	31.39 (16.66)	45.85 (46.47)	
Median(25–75%)	26.51 (21.66–35.6)	28.56 (24.21–37.63)	
TNF- α (ng/L)			0.536 *
Mean(SD)	90.24 (67.4)	137.22 (169.52)	
Median(25–75%)	74.08 (56.31–106.38)	76.16 (58.7–113.88)	
CRP (mg/L)			0.063 *
Mean(SD)	0.93 (1.21)	1.45 (1.56)	
Median(25–75%)	0.56 (0.29–1.09)	0.88 (0.66–1.5)	
TAC * (mmol/L)			0.370
Mean(SD)	1.07 (0.19)	1.02 (0.18)	
Median(25–75%)	1.02 (0.9–1.18)	1.02 (0.9–1.12)	
MDA (nmol/L)			0.224 *
Mean(SD)	8.68 (7.16)	15.15 (23.87)	
Median(25–75%)	6.61 (4.87–9.34)	7.23 (6.16–9.9)	

IL-1—Interleukin-1; IL-6—Interleukin; TNF-α—Tumor Necrosis Factor α; CRP—C-reactive protein; TAC—total antioxidant capacity; MDA—malondialdehyde. The analysis was conducted using an unpaired *t*-test (not marked) or Mann–Whitney test (*p*-value marked with an asterisk).

**Table 3:** Spearman and Pearson’s correlations among Inflammatory and Oxidative Stress markers and macronutrient intakes in Adolescent Girls with PCOS in the Ov/Ob group.

Variables	Total Protein (g)	Total Fat (g)	Total Carbohydrates (g)	Fiber (g)	Plant Protein (g)	Animal Protein (g)	SFA (g)	MUFA (g)	PUFA (g)	Total Cholesterol (mg)
IL-1 (pg/mL)										
*p* value	0.244	0.601	0.245	0.132	0.357	0.863	0.608	0.601	0.844	0.768
*r*	−0.259	0.118	−0.259	−0.331	−0.206	−0.039	0.116	0.118	−0.045	0.067
IL-6 (ng/L)										
*p* value	**0.031**	0.687	**0.037**	**0.025**	**0.007**	0.289	0.700	0.654	0.353	0.702
*r*	−0.461	−0.091	−0.448	−0.475	−0.557	−0.237	−0.087	−0.101	−0.208	−0.086
TNF-α (ng/L)										
*p* value	**0.043**	0.272	0.102	0.095	**0.006**	0.115	0.313	0.381	0.091	0.284
*r*	−0.435	−0.245	−0.357	−0.365	−0.564	−0.346	−0.225	−0.197	−0.369	−0.239
CRP (mg/L)										
*p* value	0.546	0.427	0.782	0.776	0.474	0.556	0.589	0.271	0.743	0.871
*r*	−0.136	0.178	−0.063	0.064	−0.161	−0.133	0.122	0.245	0.074	−0.037
MDA (nmol/mL)										
*p* value	0.072	0.618	**0.045**	0.052	**0.010**	0.287	0.613	0.658	0.355	0.594
*r*	−0.391	−0.112	−0.431	−0.419	−0.539	−0.238	−0.114	−0.100	−0.207	−0.120
TAC (mmol/L)										
*p* value	0.993	0.972	0.656	0.796	0.305	0.651	0.762	0.932	0.609	0.966
*r*	−0.002 *	−0.008 *	−0.101 *	−0.059	−0.229 *	−0.102 *	−0.069 *	0.019 *	0.116 *	−0.010

IL-1—Interleukin-1; IL-6—Interleukin; TNF-α—Tumor Necrosis Factor α; CRP—C-reactive protein; TAC—total antioxidant capacity; MDA—malondialdehyde. The r coefficients are either Spearman’s correlation coefficients (not marked) or Pearson’s correlation coefficients (r marked with an asterisk). All the *p*-values marked in bold were statistically significant.

**Table 4:** Spearman and Pearson’s correlation coefficients (r) between Inflammatory and Oxidative Stress markers and macronutrient intakes in the N group and Ov/Ob group.

Variables	Group	Total Protein (g)	Total Carbohydrates (g)	Fiber (g)	Plant Protein (g)	Total Cholesterol (mg)
IL-1 (pg/mL)	N	0	−0.16	0.078	0.03	0.115
	Ov/Ob	−0.259	−0.259	−0.331	−0.206	0.067
IL-6 (ng/L)	N	0.032	0.104	0.082	0.27	0.132
	Ov/Ob	**−0.461**	**−0.448**	**−0.475**	**−0.557**	−0.086
TNF-α (ng/L)	N	0.079	−0.033	0.012	0.175	0.072
	Ov/Ob	**−0.435**	−0.357	−0.365	**−0.564**	−0.239
CRP (mg/L)	N	0.058	−0.249	0.136	−0.048	**0.342**
	Ov/Ob	−0.136	−0.063	0.064	−0.161	−0.037
MDA (nmol/mL)	N	0.081	−0.045	0.028	0.218	0.193
	Ov/Ob	−0.391	**−0.431**	−0.419	**−0.539**	−0.12
TAC (mmol/L)	N	−0.199	−0.305	0.204	**−0.38**	0.067
	Ov/Ob	−0.002 *	−0.101 *	−0.059	−0.229 *	−0.01

IL-1—Interleukin-1; IL-6—Interleukin; TNF-α—Tumor Necrosis Factor; CRP—C-reactive protein; TAC—total antioxidant capacity; MDA—malondialdehyde. All the coefficients marked in bold were statistically significant. Pearson’s correlation coefficients are marked with an asterisk; otherwise, Spearman’s correlation coefficients were used.

**Table 5:** Spearman and Pearson’s correlations among Inflammatory and Oxidative Stress markers and macronutrient intakes in Adolescent Girls with PCOS in the N group.

Variables	Total Protein (g)	Total Fat (g)	Total Carbohydrates (g)	Fiber (g)	Plant Protein (g)	Animal Protein (g)	SFA (g)	MUFA (g)	PUFA (g)	Total Cholesterol (mg)
IL-1 (pg/mL)										
*p* value	0.998	0.991	0.344	0.644	0.861	0.533	0.953	0.922	0.537	0.498
*r*	0.000	0.002	−0.160	0.078	0.030	−0.106	0.010	0.017	0.105	0.115
IL-6 (ng/L)										
*p* value	0.850	0.401	0.541	0.630	0.106	0.585	0.630	0.187	0.179	0.437
*r*	0.032	0.142	0.104	0.082	0.270	−0.093	0.082	0.222	0.226	0.132
TNF-α (ng/L)										
*p* value	0.642	0.419	0.844	0.942	0.299	0.752	0.534	0.649	0.195	0,672
*r*	0.079	0.137	−0.033	0.012	0.175	−0.054	0.106	0.077	0.218	0.072
CRP (mg/L)										
*p* value	0.735	0.636	0.137	0.423	0.779	0.521	0.622	0.803	0.704	**0.038**
*r*	0.058	−0.080	−0.249	0.136	−0.048	0.109	0.084	−0.042	−0.065	0.342
MDA (nmol/mL)										
*p* value	0.632	0.605	0.792	0.871	0.196	0.741	0.725	0.408	0.250	0.252
*r*	0.081	0.088	−0.045	0.028	0.218	0.056	0.060	0.140	0.194	0.193
TAC (mmol/L)										
*p* value	0.237	0.404	0.066	0.226	**0.021**	0.153	0.587	0.329	0.076	0.693
*r*	−0.199	−0.141 *	−0.305	0.204	−0.380	−0.240	0.092 *	−0.165	−0.296	0.067

IL-1—Interleukin-1; IL-6—Interleukin; TNF-α—Tumor Necrosis Factor α; CRP—C-reactive protein; TAC—total antioxidant capacity; MDA—malondialdehyde. The r coefficients are either Spearman’s correlation coefficients (not marked) or Pearson’s correlation coefficients (r marked with an asterisk). All the *p*-value marked in bold were statistically significant.

## Data Availability

The datasets generated during and/or analysed during the current study are available from the corresponding author on reasonable request.
